# The accuracy of a recombinant antigen immunochromatographic test for the detection of *Strongyloides stercoralis* infection in migrants from sub-Saharan Africa

**DOI:** 10.1186/s13071-022-05249-z

**Published:** 2022-04-23

**Authors:** Francesca Tamarozzi, Silvia Stefania Longoni, Cristina Mazzi, Eleonora Rizzi, Rahmah Noordin, Dora Buonfrate

**Affiliations:** 1 Department of Infectious Tropical Diseases and Microbiology, IRCCS Sacro Cuore Don Calabria Hospital, 37024 Negrar, Verona Italy; 2 Clinical Research Unit, IRCCS Sacro Cuore Don Calabria Hospital, 37024 Negrar, Verona Italy; 3 Institute for Research in Molecular Medicine, Universiti Sains Malaysia, 11800 Penang, Malaysia

**Keywords:** Strongyloides, Strongyloidiasis, Rapid diagnostic test, Immunochromatographic test, Recombinant antigen, Diagnostic study

## Abstract

**Background:**

Strongyloidiasis, a nematode infection which is mainly caused by *Strongyloides stercoralis* in humans, can lead to a fatal syndrome in immunocompromised individuals. Its diagnosis is challenging due to the absence of a diagnostic gold standard. The infection is highly prevalent in migrants from endemic countries in tropical and subtropical areas, and a rapid diagnostic test would be helpful for screening purposes. The aim of this study was to estimate the accuracy of a novel immunochromatographic test (ICT) for the diagnosis of *S. stercoralis* infection.

**Methods:**

A single-centre diagnostic accuracy study was undertaken using well-characterized frozen sera available from the biobank of a referral hospital for parasitic diseases in Italy. The included sera were from migrants from sub-Saharan Africa, and matching results were available for agar plate culture and/or polymerase chain reaction for *S. stercoralis*; moreover, the results of both a commercial enzyme-linked immunosorbent assay test and an in-house immunofluorescence test for strongyloidiasis were made available. Laboratory staff who read the ICT results were blinded as regards the results of the other tests. Two readers independently read the ICT, and a third one was involved when results were discrepant. The accuracy of the ICT was assessed both against the results of the panel of faecal tests and by latent class analysis (LCA).

**Results:**

Agreement between the readers was excellent [Cohen’s κ = 92.7%, 95% confidence interval (CI) 88.3–97.1%]. When assessed against the results of the faecal tests, the sensitivity and specificity of the ICT were 82.4% (95% CI 75.7–89.0%) and 73.8% (95% CI 66.8–80.9%), respectively. According to the LCA, the sensitivity and specificity were 86.3% (95% CI 80.1–92.5%) and 73.9% (95% CI 67.0–80.8%), respectively.

**Conclusions:**

The results of the ICT demonstrated ease of interpretation. The accuracy proved good, though the sensitivity might be further improved for screening purposes.

**Graphical Abstract:**

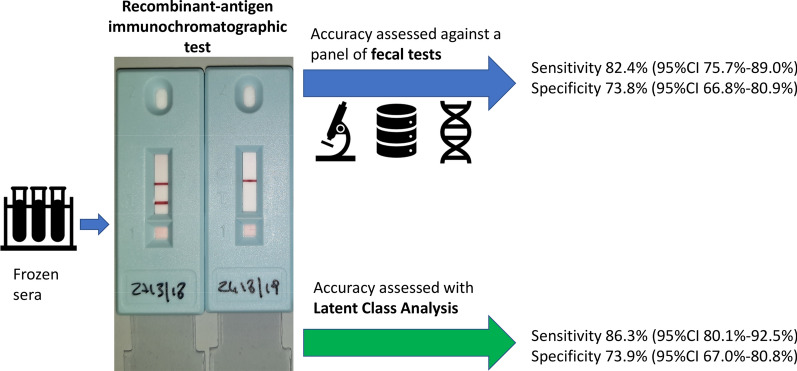

**Supplementary Information:**

The online version contains supplementary material available at 10.1186/s13071-022-05249-z.

## Background

Strongyloidiasis is a neglected tropical disease (NTD) primarily caused by the soil-transmitted helminth (STH) *Strongyloides stercoralis* [1]. According to recent estimates, around 600 million people are infected with *S. stercoralis* worldwide, particularly in Southeast Asia, Africa and the Western Pacific Region [1]. The infection causes morbidity in the affected populations and can lead to death in immunocompromised individuals [2, 3]. Based on these outcomes, the World Health Organization (WHO) recently recommended implementing control measures for strongyloidiasis in endemic areas, and included it among the targets of the WHO 2030 road map on NTDs [4].

A major problem in implementing control programmes for this infection is choosing a diagnostic tool for the baseline mapping and monitoring of interventions, since there is no gold standard for diagnosing strongyloidiasis [2]. In particular, tests widely used for the detection of other STHs (namely, *Trichuris trichiura*, hookworm and *Ascaris lumbricoides*), such as direct stool microscopy, Kato-Katz (the latter being recommended by the WHO for assessing and monitoring STH prevalence in the context of control programmes), and formol-ether concentration, have an exceedingly low sensitivity for *S. stercoralis* [5]. More sensitive coproparasitological techniques such as the Baermann method or agar plate culture (APC) are more labour intensive and time-consuming, and also require personnel skilled in their use and the microscopical diagnosis of *S. stercoralis.* Serologic and molecular methods have been deployed to achieve better sensitivity, but are not applicable in all situations due to cost constraints and the need for specific laboratory equipment and trained personnel, which are not always available in endemic areas [5, 6]. Moreover, globally there is little awareness about this NTD, hence its misdiagnosis is seldom perceived as a problem by laboratory staff and health care workers.

A rapid and easy-to-use test would help in the implementation of screening programmes for strongyloidiasis, and increase case detection, in contexts where these are most required, such as in endemic areas and for health care services for migrants in non-endemic countries. A systematic review indicated that the proportion of migrants with strongyloidiasis is not negligible [7] but, again, the lack of awareness of this NTD and the use of inadequate diagnostic methods result in its underdiagnosis.

Recently, the Institute for Research in Molecular Medicine (INFORMM) of the Universiti Sains Malaysia (Penang, Malaysia) developed an immunochromatographic test (ICT) for the detection of immunoglobulin G4 (IgG4) against *S. stercoralis*, which showed promising results [8]. This ICT has been converted from a dipstick test to a sturdier and easy-to-use cassette format, with the sensitivity and specificity of the former used as references in the development of the latter. Preliminary evaluation of the cassette showed that its accuracy is good, with a sensitivity and specificity of 97% and 94.5%, respectively [9].

The aim of this study is to estimate the accuracy of the ICT cassette developed by INFORMM for the diagnosis of strongyloidiasis in migrants from sub-Saharan Africa. The patients attended the Department of Infectious Tropical Diseases and Microbiology (DITM) of IRCCS Sacro Cuore Don Calabria Hospital, a referral centre for parasitic infections in Italy.

## Methods

This single-centre diagnostic accuracy study was carried out using well-characterized frozen sera available from the DITM Tropica biobank. The inclusion criteria were as follows: (i) the sera had been donated for research purposes by migrants from sub-Saharan Africa following routine analyses; (ii) matching results for APC and/or polymerase chain reaction (PCR) were available for *S. stercoralis*; and (iii) results from both a commercial enzyme-linked immunosorbent assay (ELISA) and an in-house immunofluorescence test (IFAT) for strongyloidiasis routinely performed at DITM (see below) were available. When available, the result of the microscopic examination of formol-ether concentrated faeces was also reported in the study database. Exclusion criteria were as follows: (i) treatment with ivermectin in the previous 6 months; and (ii) the impossibility of executing the ICT, for any reason.

All available serum samples in the biobank were considered eligible for inclusion in the study. Consecutive samples that met the inclusion criteria were included in the study until the required sample size was achieved. The results of this study are reported following the Standards for Reporting of Diagnostic Accuracy Studies checklist [10].

### Index test

The ICT is a prototype lateral flow assay that detects IgG4 against *S. stercoralis* [9]. The test line is lined with the recombinant antigen NIE [11]. After thawing, 35 µl of serum is added to the bottom square well. The serum flows up the strip to the control area; three drops of a buffer are then added to the oval top well. After 15 min, the result is visible on the cassette pad. A positive control line should be observable to verify that the test is valid. The presence of the test line in addition to the control line defines a positive result. The procedure for the implementation of this test has been described in detail previously [9].

### Tests used for the evaluation of the index test

Due to the absence of a diagnostic gold standard, the results of multiple tests for *S. stercoralis* were used to estimate the accuracy of the ICT, as explained below.

### Faecal tests

Both APC and PCR for *S. stercoralis* are routinely used at the study site. For the APC, 30 g of faeces mixed with charcoal is cultured on 10 agar plates (3 g faeces per plate). The plates are incubated at 26 °C for 48 h, followed by macroscopic examination. The plates are then washed with 2.5% formalin, which is centrifuged, and the sediment examined microscopically. In a previous retrospective study, the sensitivity of the APC method performed at the DITM was 45.4% [95% confidence interval (CI) 30.4–61.1] [12].

The real-time PCR assay is based on the method of Verweij et al. [13]. Briefly, for DNA extraction, about 200 mg of faeces is suspended in 200 µl of phosphate-buffered saline containing 2% polyvinylpolypyrrolidone (Sigma-Aldrich) and frozen overnight at − 20 °C until the extraction is performed. After thawing and boiling, the samples are processed by an automated extraction system (Magna Pure LC.2; Roche). The real-time PCR assay is performed as described previously [13]. The amplification target is the small-subunit ribosomal RNA gene sequence of *S. stercoralis*. Appropriate positive and negative controls are included in all runs. The PhHV-1 control DNA is amplified with the appropriate primers/probe mix in the same PCR reaction as the control for PCR inhibitors and amplification quality. The thermocycling comprises 40 cycles, and cycle threshold values < 40 are considered positive. The reactions, detection and data analysis are performed with the CFX96 detection system (Bio-Rad Laboratories). In a previous retrospective study, the method demonstrated a sensitivity of 56.8% (95% CI 41.0–71.6) [12].

### Serologic tests

The IFAT used in this work was an in-house method implemented at the DITM, based on antigens retrieved from *S. stercoralis* filariform larvae obtained from positive APC. The detailed procedure has been described previously [14]. A positive result is defined as a titre ≥ 1:20. A previous retrospective study demonstrated good accuracy of the test, with a sensitivity of 94.6% (95% CI 90.7–98.5) and a specificity of 87.4% (83.4–91.3) [15]. It is routinely used for screening and individual diagnosis at the study site.

The commercial ELISA was an ELISA assay based on *Strongyloides ratti* antigens, and was developed by Bordier Affinity Products. A previous retrospective study estimated its sensitivity and specificity as 90.8% (95% CI 85.8–95.7) and 94.0% (95% CI 91.2–96.9), respectively [15]. The test was performed as per the manufacturer’s instructions. However, as the cut-off varies between runs, we used a normalized optical density ratio to compare the results obtained in different sessions. A ratio ≥ 1 defines a positive result. The test is widely available and deployed for routine screening and diagnostic activities across Europe.

### Study procedures

One investigator retrieved the sera from the biobank and re-coded them. The laboratory staff performing and reading the ICT were blinded to the results of the previous reference tests. After the test had been performed by one member of laboratory staff, the ICT was read independently by two operators. The two readings were then given to an investigator not involved in the execution of the test, who sought an independent reading from a third member of laboratory staff in cases where there was disagreement between the readings of the first two operators. A test result was considered final when there was agreement on it between at least two readers. The results were reported as positive or negative. If the control band was not present on a test, the result was considered invalid and the test was repeated. In the case of a second invalid test, the final result was reported as such.

### Statistical analysis

#### Primary endpoint

The accuracy of the ICT was first assessed against the results of the faecal tests that matched each serum sample. The faecal tests were classified as positive or negative, as reported in the laboratory records corresponding to the sampling time point. In detail, the result of the faecal test panel was classified as positive in cases where at least one of the faecal tests available for that sample (formol-ether concentrated, APC, PCR) was positive. The panel result was considered negative in cases where all the available faecal tests for that sample were negative.

#### Secondary endpoint

Since there is no gold standard for the detection of *S. stercoralis*, the accuracy of the index test was also assessed by latent class analysis (LCA) [16, 17], which combined the results of the faecal and serological tests that were already available.

The ICT results were classified as positive or negative. Indeterminate results were not used for the calculation of accuracy [18]. Cohen’s κ [19] was used to assess the agreement between readers. Estimates were reported along with the 95% CI. For the primary and secondary endpoints, the test results were displayed in contingency tables from which the sensitivity and specificity were calculated. LCA is a statistical method that uses a probabilistic model to estimate the unobserved true disease status of patients (positive or negative) based on the results of observed tests that are considered imperfect classifiers of disease status. For this study, four imperfect tests were used, with the assumption of conditional independence. The data analysis was performed using SAS software, version 9.4 (SAS Institute, Cary, NC). The level of statistical significance level was set at 0.05.

#### Sample size

In Yunus et al. [8], the sensitivity and specificity of the ICT by INFORMM were 91.3% and 100.0%, respectively. A sample size of 160 produced a two-sided CI with a width equal to 0.10 when the sample sensitivity was 0.90. For specificity, the sample size was 54, assuming a specificity of 0.99 with a width equal to 0.10. As an undefined number of samples were classified as positive or negative only at the moment of data analysis (following application of the LCA, as described above), 60 additional samples were analysed to reasonably meet the expectation that the minimum target number of positive cases had been identified.

## Results

**Fig. 1:**
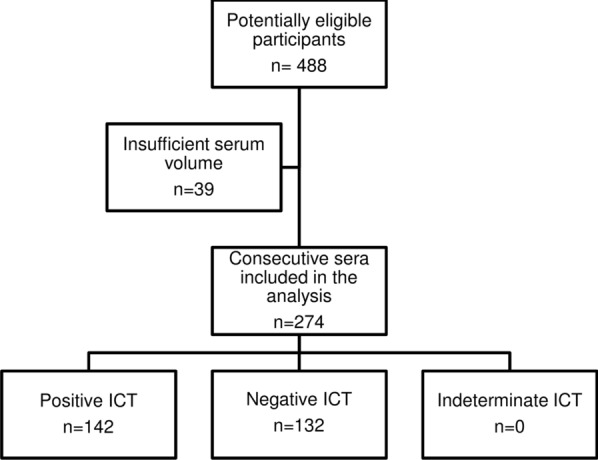
Study flow chart.* ICT* Immunochromatographic test

The study flow chart is reported in Fig. 1, which also shows the number of samples with results available for each index test. The ICT was performed on 274 serum samples, and all the results were deemed valid. The third reader was involved in 10 cases; in all of these, the discrepant results were between a negative and a faint positive result. Therefore, the agreement between readers was excellent (Cohen’s κ = 92.7%, 95% CI 88.3–97.1%).

The results of the ICT in relation to the results of the faecal tests are reported in Table 1.

**Table 1:** Immunochromatographic test (ICT) results shown against the results of the panel of faecal tests

	Faecal test positive	Faecal test negative	Total
ICT positive	103	39	142
ICT negative	22	110	132
Total	125	149	274

The sensitivity and specificity of the ICT were 82.4% (95% CI 75.7–89.0%) and 73.8% (95% CI 66.8–80.9%), respectively. The results of the other serologic tests (ELISA and IFAT) in comparison to the panel of faecal tests, and related sensitivity and specificity values, are presented in Additional file 1: Table S1.

When the LCA was applied, the results were re-classified into positive/negative categories (Table 2); based on this analysis, the ICT showed a sensitivity of 86.3% (95% CI 80.1–92.5%) and specificity of 73.9% (95% CI 67.0–80.8%).

**Table 2:** ICT results according to the latent class analysis (*LCA*)

	LCA positive	LCA negative	Total
ICT positive	101	41	142
ICT negative	16	116	132
Total	117	157	274

Co-infections with other helminths, which could possibly cause cross-reactions, were evaluated for the subgroup of sera with positive ICT and negative LCA; the results are reported in Table 3.

**Table 3:** Helminth infections in cases with positive ICT and negative LCA

Parasite	Diagnostic method	Frequency (% positive out of samples tested)
Filarial worms	Serology^a^	4 (9.8)
*Schistosoma* spp.	Serology^b^	5 (12.2)
*Schistosoma* spp.	Stool microscopy	2^c^ (4.9)
Hookworm	Stool microscopy	5 (12.2)
*Strongyloides fuelleborni*	Stool microscopy	1 (2.4)

^a^*Acanthocheilonema vitae* immunoglobulin G (IgG) enzyme-linked immunosorbent assay (ELISA; Bordier Affinity Products); a pan-filarial seroassay exploiting cross-reaction between *A. vitae* and filarial species infecting humans. Cross-reactions with other worms may also occur

^b^*Schistosoma mansoni* IgG ELISA (Bordier Affinity Products)

^c^The two patients with positive microscopy were also positive by serology for *Schistosoma*

Of note, the sample containing *Strongyloides fuelleborni* eggs was IFAT and ELISA negative.

## Discussion

In this study, we evaluated the use of a novel ICT cassette to diagnose strongyloidiasis using stored samples from migrants attending a referral centre for tropical diseases in Italy. Although the study was conducted on frozen serum samples, these had been well-characterized previously, and represent a cohort of patients for whom the test would be used.

The ICT results were easy to interpret, as demonstrated by the high agreement between readers. When the results were assessed against the panel of faecal tests, we observed that the ICT missed 22 cases, which was reduced to 16 missed cases when re-classification through LCA was applied. The specificity was also suboptimal according to the LCA, which assessed the uncertainty in the classification of positives/negatives due to the lack of a diagnostic gold standard [17]. When compared with the other serologic assays in terms of performance against the faecal test panel, the ELISA showed better sensitivity (90.4%) and slightly worse specificity (72.5%), while the IFAT (at the 1:20 dilution positivity cut-off) was the test with the highest sensitivity (95%) and worst specificity (40.9%). Based on the faecal test panel, *Strongyloides* infection is highly prevalent in the study population (45.6%). It is thus expected that a significant number of people who were negative according to the faecal panel were positive according to the serology and may harbour the infection; this may explain the low specificity shown by all three serological tests.

It should also be taken into consideration that, in the case of both the ELISA and the IFAT, previously assessed threshold values that increase their specificity indicated that an *S. ratti* ELISA with a normalized optical density > 2 and an IFAT with a > 1:160 titer should be considered almost 100% specific [15]. However, at the same time, the increased cut-off values decrease the sensitivity of the tests. Although the results of these assays are often reported qualitatively, thresholds can be useful in clinical practice, as they indicate the probability of a true positive case. Of course, this evaluation cannot be done in the case of tests that provide a qualitative result, such as the ICT.

The cassette is preferable to the dipstick because it is sturdier and its use does not require additional laboratory equipment, and it can also be used on whole blood from a finger prick. Preliminary evaluation of the use of the cassette for whole blood showed that it performed well [9], but more data are needed to estimate its accuracy. In a previous study evaluating the diagnostic performance of the cassette ICT against sera from various origins (including sera from individuals from endemic areas, individuals with proven infection, individuals with co-infection, and blood donors), the reported sensitivity and specificity were 97% and 94.5%, respectively [9]. Inconsistency between the results of the present study and those of Noordin et al. [9] might be due to the different compositions of the serum panels, which included people of different origins (only sub-Saharan Africans in the former and mostly Asians in the latter study) and the methods used to calculate accuracy. Notably, the sera used for diagnostic sensitivity and specificity in Noordin et al. [9] were not from the same populations, while in the present study, the sera were from one cohort. Furthermore, the sera used for diagnostic specificity in the former were selected from those that were negative according to a commercial ELISA kit and ICT dipstick.

A highly sensitive test is favourable in a setting where it is used as a screening method. Missing a case of a potentially fatal infection is more harmful than treating a false-positive patient with a single dose of ivermectin, which has an excellent tolerability profile [20]. Adding a second highly specific test may be useful to confirm the diagnosis and monitor the response to treatment.

The detection of IgG4 is deemed useful as a means of increasing the specificity of diagnostic tests for intestinal helminth infections [21]. However, studies comparing the IgG and the IgG4 formats of different assays for *S. stercoralis* diagnosis (including ELISAs, LIPS and another lateral flow cassette test), showed that the sensitivity of the IgG4 assay was dramatically lower than that of the IgG formats [11, 21, 22]. An ICT based on the recombinant antigen SsIR [23] showed 91.7% and 73.8% sensitivity with the IgG and IgG4 kits, respectively, while the specificity values were almost the same. Since the use of recombinant antigens is thought to produce more specific results than the use of crude antigens [11], an ICT that detects IgG would probably have the benefit of better sensitivity while retaining good specificity.

In addition to their higher specificity, recombinant antigens have other important advantages over crude antigens of *Strongyloides* larvae, i.e. they can be produced on a large scale and the results obtained with them are more reproducible. The previous dipstick version of the ICT included two recombinant antigens, NIE and Ss1a [8]. When the NIE-Ss1a dipstick and the NIE cassette formats were compared, their performance was similar to that found by Yunus et al. [8], the researchers who developed the tests (R. Noordin, personal communication). Conversely, adding SsIR antigen to a research-use-only ELISA based on NIE improved the test’s sensitivity [24].

## Conclusions

In conclusion, the ICT cassette is easy to use and the results obtained with it are easy to interpret. The sensitivity of the ICT cassette was good, but deserves further improvement if this test is to be used for screening purposes.

## Supplementary Information

**Additional file 1: Table S1.** Results of the IFAT and ELISA presented together with the results of the panel of faecal tests.

## Data Availability

The raw data will be published in a public repository upon publication of the present article.

## Abbreviations

APC: Agar plate culture
DITM: Department of Infectious Tropical Diseases and Microbiology
ELISA: Enzyme-linked immunosorbent assay
ICT: Immunochromatographic test
IFAT: Immunofluorescence test
INFORMM: Institute for Research in Molecular Medicine
LCA: Latent class analysis
NTD: Neglected tropical disease
PCR: Polymerase chain reaction
STH: Soil-transmitted helminths
WHO: World Health Organization
