# Supplementary material for: The accuracy of a recombinant antigen immunochromatographic test for the detection of Strongyloides stercoralis infection in migrants from sub-Saharan Africa
Source: Parasit Vectors. 2022 Apr 23;15:142. doi: 10.1186/s13071-022-05249-z (PMC9034504; doi:10.1186/s13071-022-05249-z)
Supplement: Supplementary file 1 — Additional file 1: Table S1. Results of the IFAT and ELISA presented together with the results of the panel of faecal tests. [file 13071_2022_5249_MOESM1_ESM.docx]

**Additional file 1: Table S1.** Results of IFAT and ELISA against the panel of faecal tests

| Serologic test | | Faecal tests positive | Faecal tests negative | Total |
| --- | --- | --- | --- | --- |
| IFAT | Positive | 119 | 88 | 142 |
|  | Negative | 6 | 61 | 67 |
| ELISA | Positive | 113 | 41 | 154 |
|  | Negative | 12 | 108 | 120 |

Sensitivity and specificity of the IFAT: 95.2% (95% CI 91.4%-98.9%) and 40.9% (95% CI 33.0%-48.8%), respectively. Sensitivity and specificity of the ELISA: 90.4% (95% CI 85.2%-95.6%), and 72.5% (95% CI 65.3%-79.6%), respectively.
